# Accumulation of long-lived mRNAs associated with germination in embryos during seed development of rice

**DOI:** 10.1093/jxb/erv209

**Published:** 2015-05-04

**Authors:** Naoto Sano, Hanako Ono, Kazumasa Murata, Tetsuya Yamada, Tadashi Hirasawa, Motoki Kanekatsu

**Affiliations:** ^1^Department of Plant Production, United Graduate School of Agricultural Science, Tokyo University of Agriculture and Technology, Fuchu, Tokyo 183-8509, Japan; ^2^Agricultural Research Institute, Toyama Agricultural, Forestry & Fisheries Research Center, Toyama, Toyama 939-8153, Japan

**Keywords:** Actinomycin D, long-lived mRNA, *Oryza sativa*, RNA-Seq, seed development, seed germination.

## Abstract

Mature dry seeds contain translatable mRNAs called long-lived mRNAs. Early studies have shown that protein synthesis during the initial phase of seed germination occurs from long-lived mRNAs, without *de novo* transcription. However, the gene expression systems that generate long-lived mRNAs in seeds are not well understood. To examine the accumulation of long-lived mRNAs in developing rice embryos, germination tests using the transcriptional inhibitor actinomycin D (Act D) were performed with the *Japonica* rice cultivar Nipponbare. Although over 70% of embryos at 10 days after flowering (DAF) germinated in the absence of the inhibitor, germination was remarkably impaired in embryos treated with Act D. In contrast, more than 70% of embryos at 20, 25, 30 and 40 DAF germinated in the presence of Act D. The same results were obtained when another cultivar, Koshihikari, was used, indicating that the long-lived mRNAs required for germination predominantly accumulate in embryos between 10 and 20 DAF during seed development. RNA-Seq identified 529 long-lived mRNA candidates, encoding proteins such as ABA, calcium ion and phospholipid signalling-related proteins, and HSP DNA J, increased from 10 to 20 DAF and were highly abundant in 40 DAF embryos of Nipponbare and Koshihikari. We also revealed that these long-lived mRNA candidates are clearly up-regulated in 10 DAF germinating embryos after imbibition, suggesting that the accumulation of these mRNAs in embryos is indispensable for the induction of germination. The findings presented here may facilitate in overcoming irregular seed germination or producing more vigorous seedlings.

## Highlight

Long-lived mRNAs stored in dry seed are translated after imbibition for germination. We report accumulation of long-lived mRNAs in developing rice embryos and candidates of the mRNAs required for germination.

## Introduction

During seed development, various macromolecules, such as carbohydrates, lipids, proteins, and mRNA, accumulate in embryos. Some of these stored molecules play important roles in seed maturation, desiccation and dormancy, but others function in the seed germination process, in which quiescent embryos rapidly derepress genetic activity following imbibition. Early studies on gene expression in germinating cotton seeds revealed that transcription is not required for *de novo* protein synthesis in imbibed seeds, suggesting that protein synthesis during the initial phase of germination is initiated from mRNA templates stored in mature dry seeds (Dure and Waters, 1965; Waters and Dure, 1966). Consistent with these reports, treatment of seeds from the model plant *Arabidopsis thaliana* and rice with a translational inhibitor has been shown to impair germination, whereas treatment of seeds with a transcriptional inhibitor had no marked effects (Rajjou *et al.*, 2004; Sano *et al.*, 2012). Taken together, these findings indicate that the translation of stored mRNA molecules in plant seeds plays an important role in the germination process.

Some of stored mRNAs in mature dry seeds are called ‘long-lived mRNAs’ because they remain translatable for long periods, even if the seeds experience stress. Numerous long-lived mRNA species are present in mature dry seeds (Rajjou *et al.*, 2004; Sano *et al.*, 2012), suggesting that transcription of these mRNAs occurs during seed development. It has been postulated that long-lived mRNAs allow for the rapid resumption of metabolic activity during seed germination (Brooker *et al.*, 1978; Martin and Northcote, 1981). In order to overcome the problem of irregular seed germination and produce more vigorous seedlings, it is important to clarify the molecular mechanisms involved in germination mediated by long-lived mRNAs. However, there are still many unsolved questions concerning the regulation machinery of long-lived mRNAs, and only a few studies have examined the significance of long-lived mRNA accumulation in developing seeds.

Seed development in rice, which is an important staple food crop in Asia, has been extensively studied. Upon fertilization, rice egg cells undergo rapid cell division and differentiation, leading to formation of the embryo. The morphogenetic events associated with embryogenesis are essentially completed ~10 days after flowering (DAF) (Itoh *et al.*, 2005; Zi *et al.*, 2013). Therefore, embryos separated from seeds harvested at 10 DAF continue to grow and will germinate after imbibing water (Still *et al.*, 1994). However, it remains unclear if embryos at 10 DAF contain sufficient long-lived mRNAs or if *de novo* gene transcription is required for germination.

Another point to be clarified regarding long-lived mRNAs in seeds is the identity of the specific mRNAs required for germination. Microarray analysis has revealed that more than 17 000 different species of stored mRNAs are present in mature dry rice embryos (Howell *et al.*, 2009). However, it is likely that not all of the stored mRNAs are translated as long-lived mRNAs during the germination process, as many are possibly involved in vital activities in cells or remain from embryogenesis processes. To identify the long-lived mRNAs that are required for germination, the transcriptome of developing embryos should be compared before and after the completion of transcription that is indispensable for germination. Currently, microarray and RNA sequencing (RNA-Seq) analyses using next generation sequencing technology are the major methods for transcriptomic analysis. According to the latest annotation of the rice genome (Os-Nipponbare-Reference-IRGSP ver. 7; http://rice.plantbiology.msu.edu/), a total of 66 338 transcripts are estimated to be transcribed from the rice genome. Although current microarray techniques do not allow for the simultaneous detection of all 66 338 of these mRNA transcripts, an RNA-Seq-based approach involving the shotgun sequencing of millions of short cDNA reads that are then mapped to a reference genome is feasible. Moreover, RNA-Seq analysis is able to accurately quantify the levels of very low and high abundance transcripts with much greater sensitivity than can be achieved using microarray-based approaches (Lu *et al.*, 2010; Mizuno *et al.*, 2010). Due to these advantages, RNA-Seq-based approaches are being increasingly used for transcriptome studies in rice (Xu *et al.*, 2012; Gao *et al.*, 2013; Zhai *et al.*, 2013). However, RNA-Seq has not been applied to a series of developing embryos between 10 DAF and 40 DAF (mature dry stage) in rice.

Here, to analyse the temporal accumulation of long-lived mRNAs required for germination in rice, we performed germination tests for developing embryos of rice treated with a transcriptional inhibitor. Two *Japonica* rice cultivars, Nipponbare and Koshihikari, were used as materials and compared in this study in order to detect the fundamental long-lived mRNAs for germination. Nipponbare is a standard *Japonica* cultivar, which has been used for the International Rice Genome Sequencing Project (Sasaki and Burr, 2000), while Koshihikari is the most commonly grown *Japonica* cultivar in Japan and has often been used as a parental line to develop new cultivars for improving eating quality. We also identified the candidates of long-lived mRNAs that are essential for germination by comparing the transcriptomes of developing embryos before and after the completion of transcription for germination using an RNA-Seq technique. Furthermore, the change of candidates for long-lived mRNAs was analysed by real-time RT-PCR using immature embryos germinating after imbibition to confirm that their accumulation is indispensable for the induction of germination.

## Materials and methods

### Plant materials and sampling

Rice plants (*Oryza sativa* L. cvs. Nipponbare and Koshihikari) were cultivated during rice growing season (May to September in 2011) under natural conditions in Toyama, Japan (36°37′N, 137°14′E). Spikelets were tagged on the day of female anthesis, and were harvested at 10, 15, 20, 25, 30 and 40 DAF. The seeds harvested at 40 DAF were air-dried and stored for 7 months at room temperature, and were then used as mature dry seeds for germination assays. The other harvested developing seeds were immediately used for germination assays without air-drying treatment.

### Germination assays

Developing embryos were separated from the dehulled seeds collected at 10, 15, 20, 25 and 30 DAF and from the mature dry seeds (40 DAF) using a surgical blade. The dissected embryos were incubated in distilled water with or without 200 µM Act D (Wako) at 28°C under dark conditions. Germination assays were carried out in triplicate using 50 embryos each at 3, 7, 10 and 14 days after imbibition (DAI). The imbibition solutions were replaced with fresh solution at the start of each germination assay. The significance of statistical differences in the germination rates for each treatment was examined using the Tukey–Kramer test.

### Extraction of total RNA from developing embryos

Total RNA was extracted from 20 embryos separated from the dehulled seeds using Fruit-mate for RNA Purification and RNAiso Plus (Takara Bio), according to the manufacturer’s protocol. The quantity and quality of the extracted total RNA was checked using an Agilent RNA 6000 Nano Kit and Agilent 2100 Bioanalyzer (Agilent Technologies). The extracted total RNA was stored at −80°C for later use.

### Preparation of cDNA libraries and sequencing

Approximately 4 μg of total RNA from 20 embryos was used for the construction of cDNA libraries (single replicate for each sample) using a TruSeq RNA Sample Prep Kit v2 (Illumina) according to the manufacturer’s instructions. Briefly, poly-A containing mRNA molecules were purified from the total RNA using poly-T oligo-attached magnetic beads and were then fragmented into small pieces using divalent cations under elevated temperature. First strand cDNA fragments were generated using SuperScript II Reverse Transcriptase and random primers (Invitrogen), and second strand cDNA fragments were then synthesized using DNA Polymerase I and RNase H. The end repair and 3’ end adenylation of these cDNA fragments were performed before the fragments were ligated with indexed adapter sequences. The adapter-modified DNA fragments were amplified by 15 cycles of PCR and the concentration and size distribution of the generated cDNA libraries were validated using an Agilent DNA 1000 Kit and Agilent 2100 Bioanalyzer (Agilent Technologies). The cDNA libraries were loaded onto a flow cell at a concentration of 10 pM for cluster generation using a TruSeq Single-Read Cluster Kit v2-cBot-GA and cBot (Illumina), and single-read sequencing reactions (40 cycles) were performed on a Genome Analyzer IIx (Illumina) with TruSeq SBS Kit v5-GA (Illumina), as directed by the manufacturer.

### Read mapping and assessment of transcript abundance

Assembly and mapping of the cDNA sequence reads were carried out using SeqMan Ngen software (DNASTAR). A total of 66 338 cDNA sequences (all.cdna of IRGSP version 7.0) obtained from the Rice Genome Annotation Project (http://rice.plantbiology.msu.edu/) were used as reference sequences for the mapping. The expression level of each transcript was calculated as reads per kilobase of exon model per million mapped reads (RPKM) (Mortazavi *et al.*, 2008) using SeqMan Ngen software (DNASTAR).

### Gene ontology analysis

GO analysis and GO enrichment was performed using the web-based tool Singular Enrichment Analysis of agriGO (http://bioinfo.cau.edu.cn/agriGO/) with ‘*Oryza sativa* TIGR5 nonTE’ as a reference background. Statistical significance of GO terms was determined by Fisher’s exact test and the Yekutieli (false-discovery rate under dependency) multi-test adjustment method with the default parameters (Du *et al.*, 2010).

### Real-Time RT-PCR analysis

Total RNA was extracted from freshly harvested 10 DAF embryos and germinating 10 DAF embryos at 7 DAI using the same method described above. A PrimeScript RT reagent kit with gDNA Eraser (Takara Bio) and an ABI 2720 Thermal Cycler (Applied Biosystems) were used to prepare cDNA from 500ng of each total RNA sample. Real-time RT-PCR analysis was performed using KAPA SYBR FAST qPCR Kit Master Mix (2X) Universal (Kapa Biosystems) and Eco Real-Time PCR System (Illumina). The thermal cycling conditions were: 95°C for 5min followed by 40 cycles of 10 s at 95°C for denaturation, and 30 s at 60°C for annealing and extension. Primers for real-time RT-PCR were designed from the target gene sequences using Primer3 software (http://frodo.wi.mit.edu) and are listed in Supplementary Table S1. The constitutively expressed gene *Actin 1* (AK100267, Li *et al.*, 2010) was used as an internal standard. Three separate experiments were performed to ensure the reliability of the results.

## Results

### Germination ability of developing embryos at 10 and 15 DAF

**Fig. 1.**
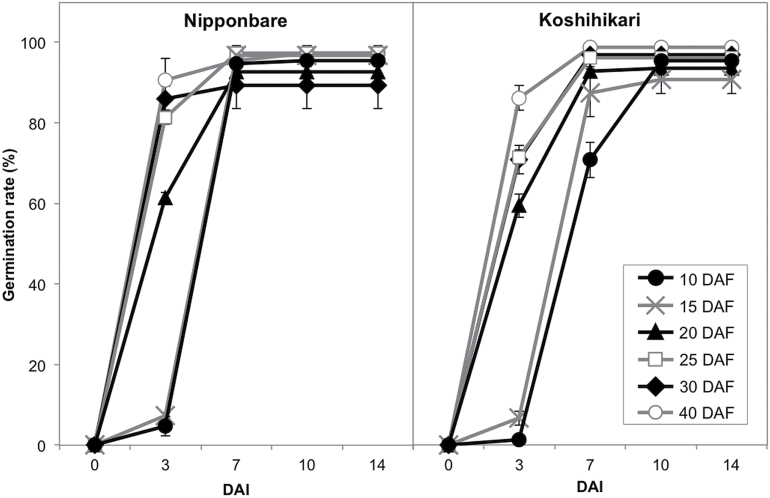
Germination rates of 10 to 40 DAF embryos. Germination vigor of developing embryos of Nipponbare and Koshihikari was evaluated at 0, 3, 7, 10 and 14 DAI. The germination rate values are presented as the means ±SE of three replicates.

To assess the germination ability of developing rice embryos, germination assays for embryos of Nipponbare dissected from seeds at 10, 15, 20, 25, 30 and 40 DAF were performed. At 7 DAI, the germination rate of all developing embryos was over 80% (Fig. 1), demonstrating that embryos of Nipponbare are able to germinate after 10 DAF. On the other hand, at 3 DAI, 80% of the 25–40 DAF embryos germinated, whereas <10% of 10 and 15 DAF embryos germinated. Similar results were also obtained for the cultivar Koshihikari. At 3 DAI, developing embryos of Koshihikari from 25–40 DAF showed >70% germination, whereas 10 and 15 DAF embryos had a germination rate of <10% (Fig. 1). Thus, the germination for 10 and 15 DAF embryos required a longer induction period compared with that of mature embryos, indicating that rice embryos before 15 DAF lack specific components required for rapid germination.

### Transcriptional inhibition impairs the germination of 10 and 15 DAF embryos

**Fig. 2.**
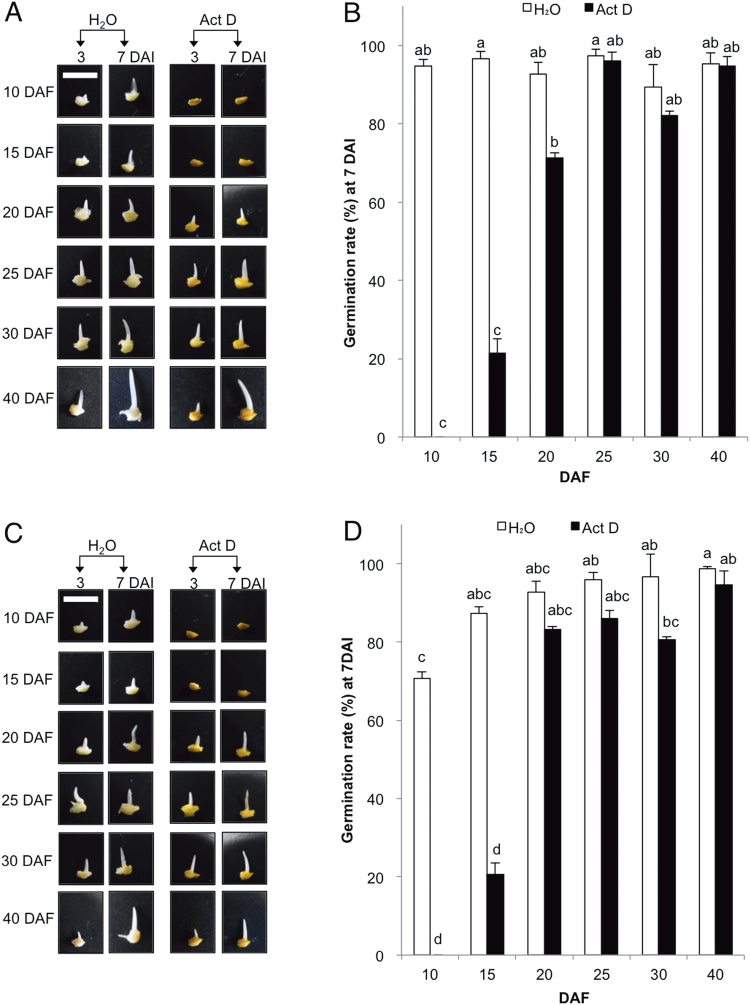
Inhibition of the germination of 10 and 15 DAF embryos by actinomycin D (Act D). Photographs show germination of developing embryos of Nipponbare (A) and Koshihikari (C) at 3 and 7 DAI with water in the presence or absence of Act D. Bar, 5mm. The germination rate values for Nipponbare (B) and Koshihikari (D) embryos are presented as the means ±SE of three replicates determined at 7 DAI with or without Act D. Identical letters represent significant difference at *P*<0.01 (Tukey–Kramer test).

To investigate the temporal accumulation of long-lived mRNAs involved in germination, the effect of the transcriptional inhibitor actinomycin D (Act D) on the germination rate of developing embryos was analysed (Fig. 2). We previously reported that *de novo* transcription in mature dry embryos of rice is suppressed during germination by treatment with 200 µM Act D (Sano *et al.*, 2012), which incorporates into double-stranded DNA template and inhibits transcription (Goldberg and Friedman, 1971). Here, 20–40 DAF embryos of Nipponbare soaked in water containing either 0 or 200 µM Act D exhibited similar germination rates under both conditions (Fig. 2A, B). In contrast, the germination of 10 and 15 DAF embryos was clearly suppressed by treatment with Act D (Fig. 2A). At 7 DAI, 10 and 15 DAF embryos of Nipponbare treated with Act D exhibited significantly inhibited germination rates (Fig. 2B; *P*<0.05, Tukey–Kramer test). Moreover, in germination assays with developing embryos of cultivar Koshihikari, a similar effect of Act D on germination was observed (Fig. 2C, D). In 10 and 15 DAF embryos treated with 200 µM Act D, germination was significantly suppressed, whereas the germination of Act D-treated 20–40 DAF embryos was not severely impaired. We also performed the germination test for developing embryos of Nipponbare grown in another location in the next year (Tokyo, 2012) and confirmed that the germination of 10 and 15 DAF embryos was substantially suppressed by treatment with Act D (Supplementary Fig. S1). These data indicate that the germination of developing embryos at 10 and 15 DAF requires *de novo* transcription, but embryos after 20 DAF are able to germinate without *de novo* transcription after imbibition. These findings suggest that long-lived mRNAs required for germination sufficiently accumulate in embryos by 20 DAF during seed development.

### Detection of candidates for long-lived mRNAs required for germination by RNA-Seq

The above findings suggested that the long-lived mRNAs required for germination do not accumulate at sufficient levels until 20 DAF. Consequently, the embryonic mRNAs that increase from 10 to 20 DAF and are maintained at high levels until 40 DAF in both Nipponbare and Koshihikari are possible candidates of the long-lived mRNAs required for germination in rice. To detect the mRNAs with this expression profile, RNA-Seq analyses of developing Nipponbare and Koshihikari embryos at 10, 20, 30 and 40 DAF were conducted.

A total of 117 million short reads from 8 libraries of developing embryos were obtained and mapped onto the reference sequence (all.cdna of IRGSP version_7.0) (Table 1). Of the 12–19 million reads in each library, 53.3–65.5% were mapped to single locations in the reference sequence (unique reads), whereas 21.1–37.7% were mapped to multiple locations (repetitive reads). The remaining 7.6–13.4% of the reads from each library did not match the reference sequence (rejected reads). Using the unique and repetitive reads, the abundance of each mRNA mapped to the reference sequence was estimated and reported as reads per kilobase of exon model per million mapped reads (RPKM) (Mortazavi *et al.*, 2008). Applying RPKM>1 as a cutoff value for mRNA abundance, a total of 17 126–19 707 mRNAs were detected from each library (Supplementary Fig. S2). These values were consistent with the ~17 000 mRNAs that were reportedly detected from mature dry embryos of rice seeds by microarray analysis (Howell *et al.*, 2009).

**Table 1.** Summary of read numbers based on the RNA-Seq data in the libraries of developing rice embryos

**RNA-Seq library**		**Total Sample reads**	**Mapped reads**	**Unique reads assigined**	**Repeated reads assigned**	**Reads rejected**
Nipponbare	10 DAF	15 552 216	13 929 733 (89.6%)	10 030 799 (64.5%)	3 898 934 (25.1%)	1 622 483 (10.4%)
	20 DAF	18 159 744	16 125 414 (88.8%)	11 324 573 (62.4%)	4 800 841 (26.4%)	2 034 330 (11.2%)
	30 DAF	19 812 792	18 023 315 (91.0%)	10 559 998 (53.3%)	7 463 317 (37.7%)	1 789 477 (9.0%)
	40 DAF	12 971 869	11 241 492 (86.7%)	7 501 998 (57.8%)	3 739 494 (28.8%)	1 730 377 (13.3%)
Koshihikari	10 DAF	12 708 066	11 746 618 (92.4%)	7 969 087 (62.7%)	3 777 531 (29.7%)	9 614 48 (7.6%)
	20 DAF	12 139 030	11 001 662 (90.6%)	7 124 194 (58.7%)	3 877 468 (31.9%)	1 137 368 (9.4%)
	30 DAF	12 897 882	11 357 135 (88.1%)	7 394 576 (57.3%)	3 962 559 (30.7%)	1 540 747 (11.9%)
	40 DAF	13 385 231	11 589 949 (86.6%)	8 771 401 (65.5%)	2 818 548 (21.1%)	1 795 282 (13.4%)

**Fig. 3.**
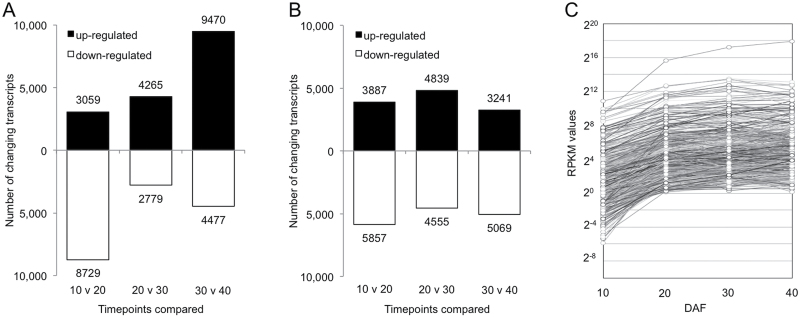
Changes in abundance of candidate long-lived mRNAs that are required for germination during rice seed development. (A, B) Number of transcripts that showed greater than 2-fold changes in abundance between successive time points of developing embryos. The numbers of up-regulated (black bar) and down-regulated (white bar) transcripts of Nipponbare (A) or Koshihikari (B) between 10 and 20 DAF, 20 and 30 DAF, and 30 and 40 DAF are summarized respectively. (C) Fluctuation of the RPKM values of 529 candidate long-lived mRNAs required for germination at 10, 20, 30 and 40 DAF. The RPKM values are the means of two cultivars (Nipponbare and Koshihikari).

To identify the specific long-lived mRNAs required for the germination of rice embryos, we compared the embryonic transcriptomes of Nipponbare and Koshihikari at successive time points during seed development, and the number of mRNAs whose RPKM values changed more than 2-fold are shown in Fig. 3A, B. From 30–40 DAF, 9470 mRNAs were up-regulated in Nipponbare, whereas only 3241 mRNAs increased in Koshihikari, suggesting that these mRNAs may be involved in varietal differences of germination between Nipponbare and Koshihikari. We also found that a number of mRNAs (8729 in Nipponbare and 5857 in Koshihikari) decreased from 10 to 20 DAF. Some of these down-regulated mRNAs may be important for morphogenesis of embryos, since the rice embryo reaches morphological maturity at ~10 DAF, and gene expressions related to cell growth or differentiation are suppressed in developing embryos after 10 DAF (Xu *et al.* 2012). On the other hand, 3059 and 3887 mRNAs increased from 10 to 20 DAF in Nipponbare and Koshihikari, respectively. Among them, 529 mRNAs were identified as potential candidates of long-lived mRNAs required for germination in developing embryos (Supplementary Table S2) by using the following stringent criteria: (i) RPKM values increased more than 2-fold at 20, 30 and 40 DAF in comparison with 10 DAF, (ii) RPKM cutoff value of >1 for gene expression at 20, 30 and 40 DAF, and (iii) mRNAs commonly detected in Nipponbare and Koshihikari. The changes in abundance of the 529 mRNAs during embryo development are shown in Fig. 3C.

### Candidates of long-lived mRNAs for germination encode proteins involved in metabolism, transport, and stress responses

**Fig. 4.**
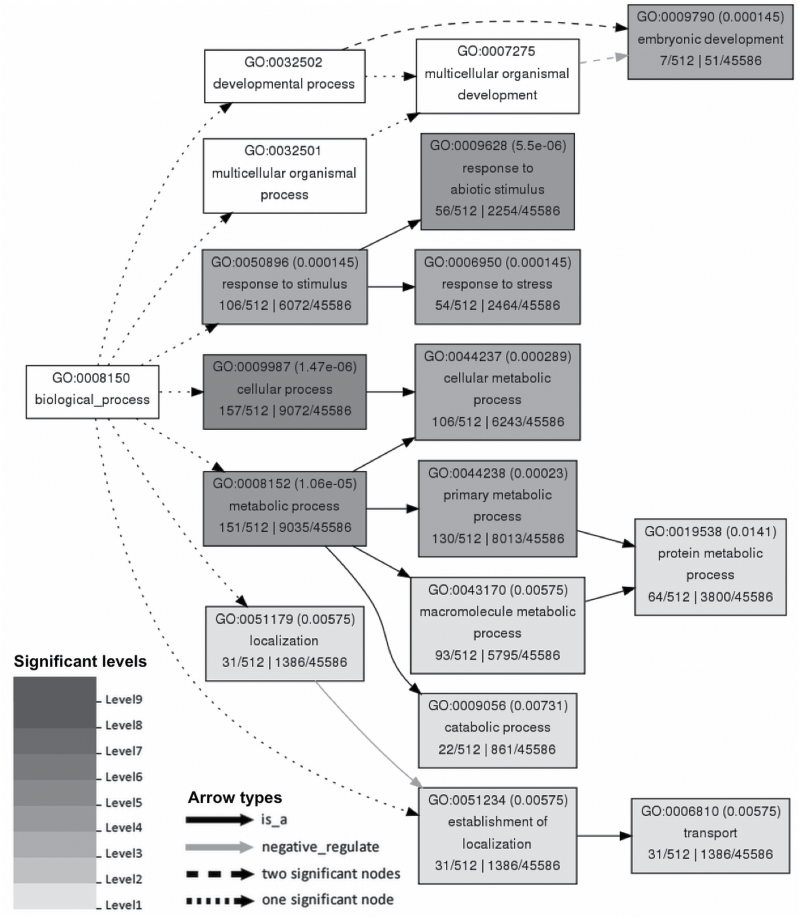
Hierarchical tree graph of overrepresented GO terms of candidates for long-lived mRNAs required for germination in the ‘biological process’ category. Boxes in the graph contain GO terms, their GO ID, and statistical information. Significant terms (FDR<0.05) are indicated by grey boxes at various significance levels, and non-significant terms are shown as white boxes. The rank direction of the graph is set from left to right.

To determine the physiological processes that are regulated by the 529 long-lived mRNA candidates during rice seed germination, Gene Ontology (GO) enrichment analysis was performed using agriGO (Du *et al.*, 2010). As 10 transcript variants were identified among the 529 long-lived mRNA candidates, 519 genes were subjected to Singular Enrichment Analysis (SEA), which identified a total of 512 annotated genes. The gene annotations (GO terms) of the 512 genes were compared with those of the 45 586 genes in the reference rice genome. The comparison revealed that 14 GO terms in the ‘biological processes’ ontology, 1 GO term in the ‘molecular functions’ ontology, and 10 GO terms in the ‘cellular components’ ontology were significantly enriched (FDR<0.05) in the 512 candidate genes of the long-lived mRNAs required for germination (Table 2). To determine which cellular processes were most critical for germination, we further generated a hierarchical tree graph of overrepresented GO terms in the biological process category (Fig. 4). The specific GO terms that were enriched among the 512 genes were ‘cellular metabolic process’, ‘protein metabolic process’, ‘transport’, ‘response to abiotic stimulus’, ‘response to stress’ and ‘embryonic development’.

**Table 2.** Significant GO terms for candidates of long-lived mRNAs required for germination

**ID**	**Ontology**	**Description**	**Input**	**Reference**	**p-value**	**FDR**
GO:0009987	Biological Process	cellular process	157	9072	6.20E-09	1.50E-06
GO:0009628		response to abiotic stimulus	56	2254	4.70E-08	5.50E-06
GO:0008152		metabolic process	151	9035	1.30E-07	1.10E-05
GO:0009790		embryonic development	7	51	3.70E-06	0.00015
GO:0050896		response to stimulus	106	6072	2.90E-06	0.00015
GO:0006950		response to stress	54	2464	3.50E-06	0.00015
GO:0044238		primary metabolic process	130	8013	6.80E-06	0.00023
GO:0044237		cellular metabolic process	106	6243	9.80E-06	0.00029
GO:0006810		transport	31	1386	0.00032	0.0057
GO:0051234		establishment of localization	31	1386	0.00032	0.0057
GO:0051179		localization	31	1386	0.00032	0.0057
GO:0043170		macromolecule metabolic process	93	5795	0.00029	0.0057
GO:0009056		catabolic process	22	861	0.00043	0.0073
GO:0019538		protein metabolic process	64	3800	0.0009	0.014
GO:0005488	Molecular Function	binding	148	9146	1.30E-06	0.0001
GO:0044464	Cellular Component	cell part	159	10811	9.90E-05	0.0059
GO:0005623		cell	161	10833	5.00E-05	0.0059
GO:0044424		intracellular part	125	8195	0.00017	0.0065
GO:0005622		intracellular	127	8397	0.00022	0.0065
GO:0043231		intracellular membrane- bounded organelle	112	7469	0.00078	0.012
GO:0043229		intracellular organelle	116	7795	0.00081	0.012
GO:0043227		membrane-bounded organelle	112	7469	0.00078	0.012
GO:0043226		organelle	116	7795	0.00081	0.012
GO:0005737		cytoplasm	105	7085	0.0017	0.023
GO:0009579		thylakoid	20	906	0.004	0.048

For the GO term ‘cellular metabolic process’, 11 *cytochrome P450* genes were detected (Supplementary Table S3), including the *OsABA8ox1* gene (Os02g47470.1), encoding an ABA 8′-hydroxylase that catabolizes abscisic acid (ABA) to phaseic acid (Saika *et al.*, 2007). The GO term ‘protein metabolic process’ included 15 genes encoding protein kinases (Os01g04460.1, Os02g12660.1, Os02g45750.1, Os03g27280.1, Os03g60150.1, Os05g25350.1, Os05g43840.1, Os06g43270.1, Os06g47470.1, Os07g35260.1, Os07g35680.1, Os07g49470.1, Os08g28710.1, Os09g27010.1 and Os10g06510.1) and five genes encoding protein phosphatase 2Cs (PP2C) (Os04g25570.1, Os05g49730.1, Os06g33549.1, Os06g48300.1 and Os09g15670.1) (Supplementary Table S4). Among the identified rice genes associated with ‘protein metabolic processes’, *CrRLK1L* (Os05g25350.1) and *PP2C* (Os09g15670.1) shared relatively high homology with *Arabidopsis thaliana Receptor-like protein kinase FERONIA* (At3g51550; 51% amino acid homology) and *ABA hypersensitive germination 3* (*AHG3*); (At3g11410; 51% amino acid homology), respectively. *FERONIA* and *AHG3* are reported to function as negative regulators in phytohormone abscisic acid (ABA) signalling (Yoshida *et al.*, 2006; Yu *et al.*, 2012), suggesting that *CrRLK1L* (Os05g25350.1) and *PP2C* (Os09g15670.1) may have a similar function in rice embryos. In addition to these protein kinases and protein phosphatase 2Cs, several genes involved in calcium ion signalling (*calmodulin-like proteins*: Os01g04330.1, Os01g59530.1, and Os04g41540.1; and *calmodulin-dependent protein kinases*: Os03g27280.1 and Os05g43840.1) and phospholipid signalling (*phosphatidylinositol-3,4 kinases*: Os04g57300.1 and Os09g29890.1) were also functionally annotated in the GO grouping of ‘protein metabolic process’ (Supplementary Table S4). Furthermore, eight *heat shock protein genes* (Os01g42190.1, Os02g43930.1, Os03g11910.1, Os03g16920.1, Os03g44620.2, Os04g01740.1, Os05g38530.1 and Os12g07060.1) were present in this functional group. Genes encoding calmodulin-like protein, calmodulin-dependent protein kinase and heat shock proteins were also detected among genes annotated by the GO terms ‘response to abiotic stimulus’ and ‘response to stress’ (Supplementary Tables S5, S6). Three genes encoding calcium ion-binding proteins (Os04g43200.1, Os04g44870.1 and Os09g24580.1) were grouped among these stress-related GO terms. Moreover, the GO term ‘transport’ included genes involved in calcium ion transport (*sodium/calcium exchanger protein*: Os03g45370.1) and phospholipid transport (*phosphatidylinositol transfer*: Os01g50616.1, and Os05g46720.1; *CRAL/TRIO domain containing protein*: Os05g18470.1) (Supplementary Table S7), whereas all genes functionally annotated as belonging to the GO term ‘embryonic development’ were *late embryogenesis abundant* (*LEA*) *protein genes* (Supplementary Table S8). Taken together, the results of the GO enrichment analyses revealed that the candidate genes of long-lived mRNAs required for germination were mainly implicated in metabolic, transport and stress response processes.

### Long-lived mRNAs involved in germination increase in 10 DAF embryos after imbibition

To examine the temporal transcriptional patterns of the identified mRNAs during rice seed germination, we examined the germination process of 10 DAF rice embryos. Although 10 DAF embryos are not capable of rapid germination, at 7 DAI, they showed more than 70% germination (Figs 1, 2). This finding suggested that long-lived mRNAs required for germination are transcribed after imbibition. GO analysis revealed that the candidate long-lived mRNAs involved in the germination process encode proteins belonging to various functional groups, and included several heat shock and LEA proteins (Supplementary Tables S4, S8). However, we previously demonstrated that several of these stress-related proteins function in the desiccation phase of seed development in rice (Sano *et al.*, 2013), and are therefore not likely specific to the germination process.

**Fig. 5.**
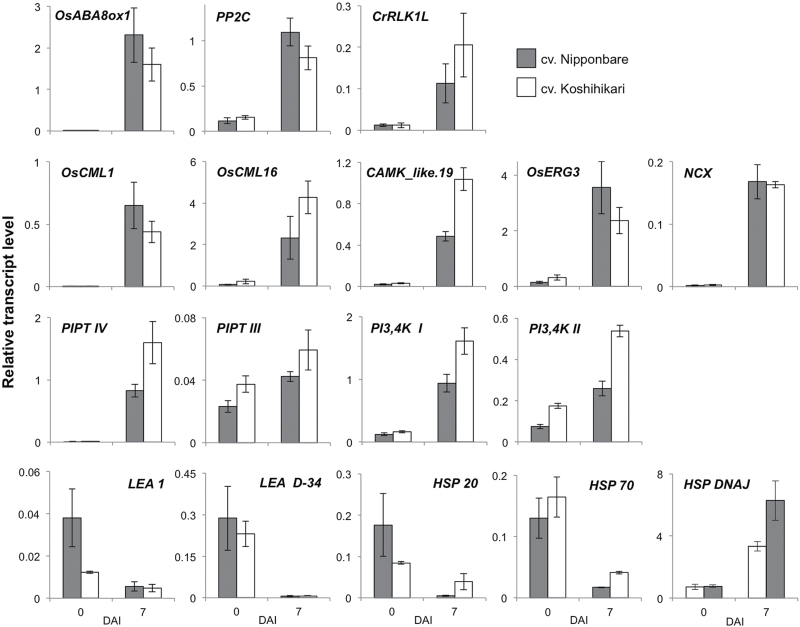
Induction of long-lived mRNA candidates required for germination in 10 DAF embryos after imbibition. Relative transcript levels of candidate long-lived mRNAs in 10 DAF embryos of Nipponbare (grey bars) and Koshihikari (white bars) at 0 and 7 DAI were analysed by real-time RT-PCR. Data are shown as the means ± SE of three replicates. *Actin 1* was used as an internal standard.

To confirm this speculation, real-time RT-PCR was to test whether the long-lived mRNAs encoding ABA, calcium ion and phospholipid signalling-related proteins, heat shock proteins and LEA proteins were induced in 10 DAF embryos of Nipponbare and Koshihikari after imbibition (Fig. 5). The mRNA transcripts of *PP2C* (Os09g15670.1), *CrRLK1L* (Os05g25350.1) and *OsABA8ox1* (Os02g47470.1) clearly increased at 7 DAI in 10 DAF embryos of Nipponbare and Koshihikari. Moreover, the transcripts of calcium ion signalling-related genes (*OsCML1*: Os01g59530.1, *OsCML16*:Os01g04330.1, *CAMK_like.19*: Os03g27280.1, *OsERG3*: Os04g44870.1 and *NCX*: Os03g45370.1) and phospholipid signalling-related genes (*PIPT IV*: Os05g46720.1, *PIPT III*: Os01g50616.1, *PI3,4K I*: Os04g57300.1 and *PI3,4K II*: Os09g29890.1) increased at 7 DAI in embryos of both cultivars. These results indicate that the long-lived mRNAs encoding ABA, calcium ion and phospholipid signalling-related proteins are present in low abundance in 10 DAF embryos, but are actively transcribed during imbibition to initiate the germination process.

The real-time RT-PCR analysis also revealed that transcripts encoding LEA proteins (*LEA 1*: Os06g02040.1 and *LEA D-34*: Os06g23350.1) and heat shock proteins (*HSP 20*: Os03g14180.1 and *HSP 70*: Os03g16920.1) were decreased at 7 DAI in both Nipponbare and Koshihikari embryos, suggesting that these mRNAs are not required for the induction of germination and that their translational products may function in responses to stress, such as desiccation during seed development, as previously reported (Sano *et al.*, 2013). Thus, we concluded that these stored mRNAs are not required for the germination of rice embryos. Surprisingly, however, mRNA transcript levels of another type of heat shock protein gene, *HSP DNAJ* (Os03g44620.2), clearly increased at 7 DAI in both Nipponbare and Koshihikari embryos, indicating that this gene is transcribed in 10 DAF embryos after imbibition and is involved in the germination process.

## Discussion

### Accumulation phase of long-lived mRNAs required for germination in developing rice embryos

**Fig. 6.**
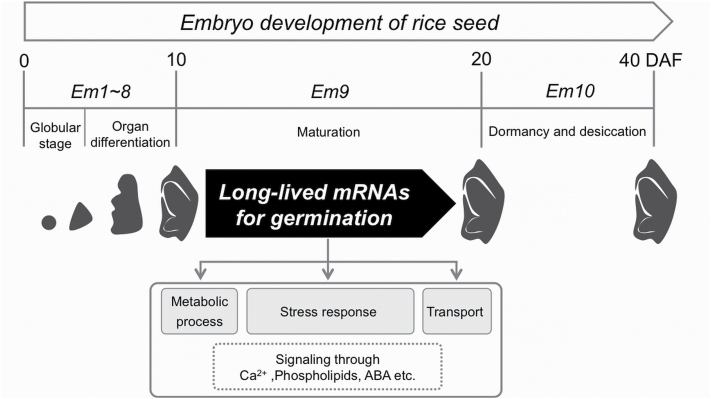
Accumulation of long-lived mRNAs required for germination in developing embryos of rice. The fertilization of an egg cell at 0 DAF initiates embryo development. Embryonic organs are formed by 10 DAF. From 10 to 20 DAF is a maturation phase in which long-lived mRNAs encoding proteins involved in metabolism, stress responses and transport processes accumulate in embryos. After 20 DAF, embryos are dormant and desiccated. The categorization of the developmental stages from Em 1 to Em 10 was proposed by Itoh *et al.* (2005).

The present study clearly demonstrates that 200 µM Act D significantly inhibits the germination of 10 and 15 DAF embryos, but has no marked effects on 20 DAF embryos (Fig. 2), indicating that long-lived mRNAs required for germination accumulate in embryos from 10 to 20 DAF. Additionally, as 10 and 15 DAF embryos require longer imbibition periods to initiate germination than 20 DAF embryos, even without Act D treatment (Fig. 1), developing rice embryos appear to acquire the ability for rapid germination from 15 to 20 DAF, which corresponds to the period when mRNAs required for germination accumulate. These results also suggest that the observed germination delay in 10 and 15 DAF embryos is due to the fact that mRNAs essential for germination are not actively transcribed until after the beginning of imbibition. We therefore conclude that the accumulation of long-lived mRNAs required for germination predominantly occurs during embryo development in rice (Fig. 6).

Itoh *et al.* (2005) categorized the development of the rice embryo into 10 stages, from ‘Em 1’ to ‘Em 10’, based on morphological analysis. According to the proposed classification scheme, Em 8 occurs at 10 DAF, when the rice embryo reaches morphological maturity. The next stage, Em 9, takes place between 11 and 20 DAF, and is proposed as a maturation phase, in which embryonic organs slightly enlarge and mRNA transcripts of LEA proteins, such as OSEM and Rab16A, increase. However, as significant morphogenetic events do not occur after stage Em 8, it has been difficult to characterize the physiological events that occur in the late stages of embryo development. Here, we showed that long-lived mRNAs required for germination predominantly accumulate in embryos between 10 to 20 DAF, indicating that rice embryos acquire the capacity for germination during the Em 9 stage (Fig. 6). The Em 10 stage, which occurs after 20 DAF, corresponds to the final stage of embryo development. In this phase, the water content of the seed declines and the embryo becomes dormant (Itoh *et al.*, 2005; Sano *et al.*, 2013), indicating that the transcription of long-lived mRNAs involved in germination is completed before the start of this desiccation/dormancy phase. However, in the present germination assay, the inhibition of germination by seed dormancy was not observed in Nipponbare or Koshihikari. This may be due to the use of dissected embryos in the germination assay, as seed dormancy can be imposed by not only the embryo, but also by the envelopes, such as the seed coat and endosperm, or through a combination of both factors (Bewley, 1997). Further analysis focusing on seed structure is required to reveal the impact of seed dormancy on long-lived mRNA levels in rice.

### Candidates of long-lived mRNAs required for germination

Using an RNA-Seq-based approach, we identified 529 mRNAs that are potentially specifically required for germination (Supplementary Table S2). On the other hand, it is known that the transcriptome profile in plants can be affected by their growth conditions, and then we analysed the changes in transcript abundance for 529 mRNAs in developing rice embryos by using the microarray gene expression data, which are publicly available (GEO DataSets, Series: GSE39432, http://www.ncbi.nlm.nih.gov/gds). Among the 529 mRNAs, 478 mRNAs could be detected from the microarray data of developing embryos in Nipponbare and revealed that most of the mRNAs are highly accumulated in 28 and 42 DAF embryos relative to in 10 DAF embryos (Supplementary Fig. S3). These results suggest that the accumulation of long-lived mRNAs identified in this study is relatively insusceptible to the plant growth conditions.

GO analysis revealed that the candidate genes of the identified mRNAs are mainly involved in metabolic, transport and stress response processes, and encode proteins such as regulators of ABA, calcium ion and phospholipid signalling, heat shock proteins and LEA proteins (Fig. 4). However, mRNAs of the LEA proteins HSP 20 and HSP 70 were not considered to be required for germination, because their levels decreased in 10 DAF embryos after imbibition (Fig. 5). In contrast, the accumulation of mRNA transcripts for ABA, calcium ion and phospholipid signalling-related genes and *HSP DNAJ* was observed in 10 DAF embryos after imbibition (Fig. 5), suggesting that these transcripts are long-lived mRNAs that play key roles in the germination process.

ABA is a phytohormone that is involved in the inhibition of seed germination. In the present study, *CrRLK1L* (Os05g25350.1) and *PP2C* (Os09g15670.1), which may negatively regulate ABA signalling in rice, and the *OsABA8ox1* gene (Os02g47470.1), encoding an ABA catabolism-related enzyme (Saika *et al.*, 2007), were identified as candidate genes of long-lived mRNAs required for germination (Supplementary Table S2). Another candidate gene detected in the RNA-Seq analysis was *OsMFT2* (Os01g02120.1), which shares 59% amino acid identity with *Mother of FT and TFL1* (At1g18100) of *Arabidopsis*. Xi *et al.* (2010) reported that Mother of FT and TFL1 promotes embryo growth through a negative feedback loop that modulates ABA signalling during seed germination, indicating that OsMFT2 may have a similar role in rice embryos. These results suggest that rice embryos store long-lived mRNAs during seed development that encode negative regulators of ABA signalling and promote seed germination after imbibition.

Phospholipid molecules are one of the main structural components of cell membranes, but have also been shown to function as second messengers that regulate plant growth and cellular responses to stress (Xue *et al.*, 2009). Phosphatidylinositiol kinases are enzymes for the regulation of phospholipid signalling in plants. Here, four transcripts encoding phosphatidylinositiol kinases (Os02g57660.1, Os02g57660.2, Os04g57300.1 and Os09g29890.1) were identified as candidates of long-lived mRNAs required for the germination of rice embryos (Supplementary Table S2). Liu *et al.* (2012) reported that phosphatidylinositol 3-kinase accelerates the process of rice seed germination through regulating NADPH oxidase activity in the plasma membrane, suggesting that the phosphatidylinositiol kinases detected in the present study may promote rice seed germination. In addition, three transcripts encoding phospholipid transfer proteins (Os01g50616.1, Os05g18470.1 and Os05g46720.1) were also identified as candidate long-lived mRNA transcripts that are required for germination (Supplementary Table S2). Phosphatidylinositol transfer proteins are crucial for lipid signalling, and Grosbois *et al.* (1989) demonstrated that the amount and activity of phospholipid transfer proteins increase during the germination process in maize seeds. Thus, these findings, when taken together with the present results, suggest that long-lived mRNA transcripts for phospholipid signalling exist in embryos before imbibition and have important roles in the induction of seed germination.

A total of 12 transcripts encoding calcium ion signalling-related proteins, including calmodulin-like proteins (Os01g04330.1, Os01g59530.1 and Os04g41540.1), calmodulin-dependent protein kinases (Os03g27280.1 and Os05g43840.1), calcium ion-binding proteins (Os04g43200.1, Os04g44870.1, Os04g51250.1, Os09g24580.1, Os09g30490.1 and Os12g06510.2) and sodium/calcium exchanger protein (Os03g45370.1), were also detected as candidates of long-lived mRNAs required for germination (Supplementary Table S2). Calcium ions are considered to be major secondary messengers and are implicated in plant growth and responses to stress or phytohormones. Yang *et al.* (2011) reported that overexpressing a calcium/calmodulin-dependent protein kinase of wheat in *Arabidopsis* plants reduced their sensitivity to ABA during seed germination. Therefore, the long-lived mRNAs encoding calcium ion signalling-related proteins identified here may participate in the induction of seed germination in rice.

In rice seeds, many types of HSPs have been detected, and it has been suggested that these proteins function in various processes, such as morphological development of embryos, desiccation in late embryogenesis, and stress response during germination (Kim *et al.*, 2009; Sano *et al.*, 2013; Zi *et al.*, 2013;). We demonstrated that transcripts of *HSP DNAJ* (Os03g44620.2) are increased both in developing embryos during the Em 9 stage (Supplementary Table S2) and in 10 DAF embryos after imbibition (Fig. 5). HSP DNA J is a J-protein/HSP 40 family protein, which are involved in *de novo* protein folding, translocation of polypeptides across cellular membranes and degradation of misfolded proteins (Craig *et al.*, 2006), suggesting that HSP DNAJ (Os03g44620.2) may promote the induction of seed germination in rice by facilitating the correct folding of proteins.

### Translations of long-lived mRNA candidates required for germination

We previously performed the proteomic analysis of embryonic proteins synthesized in germinating rice seeds treated with a transcriptional inhibitor (Sano *et al.*, 2012). By using a gel-based proteomic approach, only 20 proteins were identified as translational products of long-lived mRNAs and many of them are involved in metabolic processes such as carbohydrate metabolism. In this study, GO analysis revealed that the majority of candidate genes for long-lived mRNAs are related to metabolic processes. These suggest that some of the proteins functioning in the metabolic process during the initial phase of seed germination are synthesized from long-lived mRNAs. Han *et al.* (2014) found that many metabolic proteins are up-regulated in germinating rice embryos by using a gel free-proteomic method and for example, one of the long-lived mRNA candidate genes, *OsCML16*, annotated in the protein metabolic process in the present study, were identified as up-regulated proteins during germination. In any case, further detailed analysis focusing on the translational processes of long-lived mRNA candidates identified in the present study will be required to provide a comprehensive view on the induction of seed germination.

## Supplementary Data

Supplementary data are available at *JXB* online.

Supplementary Figure S1. Effect of Act D on the germination of developing embryos of Nipponbare cultivated in 2012.

Supplementary Figure S2. Number of transcripts in developing rice embryos.

Supplementary Figure S3. Changes in the transcript abundance of 478 long-lived mRNAs in developing embryos of Nipponbare.

Supplementary Table S1. Sequences of primer sets for real-time RT-PCR analysis.

Supplementary Table S2. Candidates of long-lived mRNAs required for germination accumulating in developing rice embryos.

Supplementary Table S3. Candidates of long-lived mRNAs required for germination corresponding to the GO term ‘cellular metabolic process’.

Supplementary Table S4. Candidates of long-lived mRNAs required for germination corresponding to the GO term ‘protein metabolic process’.

Supplementary Table S5. Candidates of long-lived mRNAs required for germination corresponding to the GO term ‘response to abiotic stimulus’.

Supplementary Table S6. Candidates of long-lived mRNAs required for germination corresponding to the GO term ‘response to stress’.

Supplementary Table S7. Candidates of long-lived mRNAs required for germination corresponding to the GO term ‘transport’.

Supplementary Table S8. Candidates of long-lived mRNAs required for germination corresponding to the GO term ‘embryonic development’.

Supplementary Data

## Abbreviations:

Act D: actinomycin D
DAF: days after flowering
DAI: days after imbibition.
