# Supplementary material for: Accumulation of long-lived mRNAs associated with germination in embryos during seed development of rice
Source: J Exp Bot. 2015 May 4;66(13):4035–46. doi: 10.1093/jxb/erv209 (PMC4473999; doi:10.1093/jxb/erv209)
Supplement: Supplementary Data [file supp_66_13_4035__index.html]

Accumulation of long-lived mRNAs associated with germination in embryos during seed development of rice — Supplementary Data 

# Accumulation of long-lived mRNAs associated with germination in embryos during seed development of rice

## Supplementary Data

Data files

**Files in this Data Supplement:**

- Supplementary Data - Supplementary Data
- Supplementary Data - Supplementary Data
