# Supplementary material for: Accumulation of long-lived mRNAs associated with germination in embryos during seed development of rice
Source: J Exp Bot. 2015 May 4;66(13):4035–46. doi: 10.1093/jxb/erv209 (PMC4473999; doi:10.1093/jxb/erv209)
Supplement: Supplementary Data [file supp_erv209_erv209_SuppFigs_new.pdf]

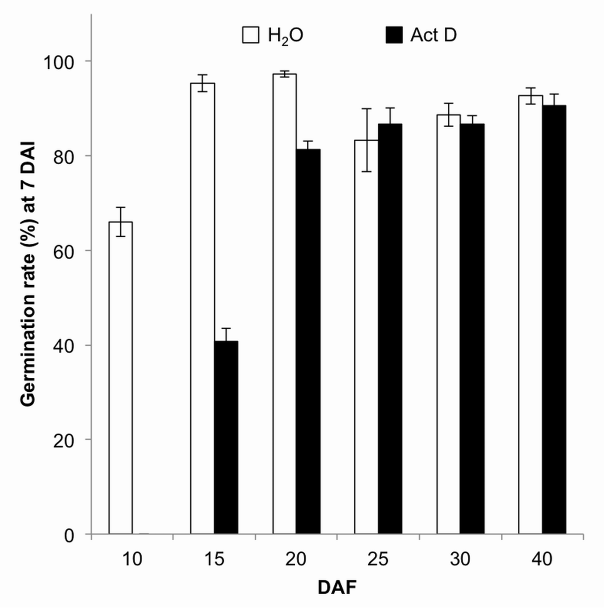


**Fig. S1.** Effect of Act D on the germination of developing embryos of Nipponbare cultivated in 2012. Rice plants (*Oryza sativa* L. cv. Nipponbare) were cultivated during rice growing season (May to September in 2012) under natural conditions in Tokyo, Japan (35°40'N, 139°28'E). The germination rate values for embryos are presented as the means ±SE of three replicates determined at 7 DAI with or without Act D.

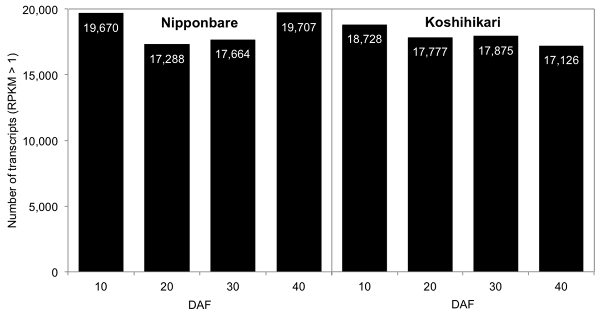


**Fig. S2.** Number of transcripts in developing rice embryos. Embryonic transcripts at 10, 20, 30 and 40 DAF in Nipponbare and Koshihikari were detected by RNA-Seq and the number was estimated using RPKM > 1 as a cutoff for gene expression values.

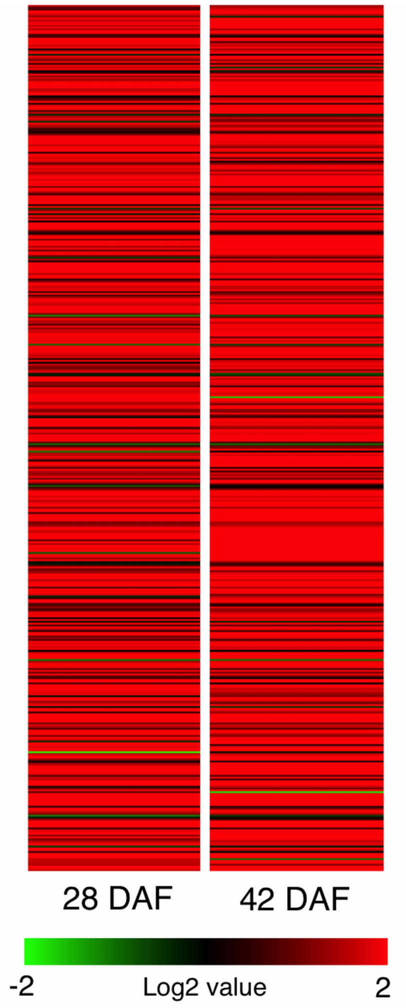


**Fig. S3.** Changes in the transcript abundance of 478 long-lived mRNA candidates in developing embryos of Nipponbare. The elements indicate the fold changes in the 478 mRNAs abundances at 28 and 42 DAF relative to at 10 DAF. The gene expression data were obtained from the microarray gene expression data which are publicly available (GEO DataSets, Series: GSE39432, http://www.ncbi.nlm.nih.gov/gds). Scale bar represents fold change (log2 value).
